# Tailoring Hydrothermal Vent Biodiversity Toward Improved Biodiscovery Using a Novel *in situ* Enrichment Strategy

**DOI:** 10.3389/fmicb.2020.00249

**Published:** 2020-02-21

**Authors:** Runar Stokke, Eoghan P. Reeves, Håkon Dahle, Anita-Elin Fedøy, Thomas Viflot, Solveig Lie Onstad, Francesca Vulcano, Rolf B. Pedersen, Vincent G. H. Eijsink, Ida H. Steen

**Affiliations:** ^1^Department of Biological Sciences, University of Bergen, Bergen, Norway; ^2^K.G. Jebsen Centre for Deep Sea Research, University of Bergen, Bergen, Norway; ^3^Department of Earth Science, University of Bergen, Bergen, Norway; ^4^Faculty of Chemistry, Biotechnology and Food Science, Norwegian University of Life Sciences (NMBU), Ås, Norway

**Keywords:** deep sea, hydrothermal sediments, *in situ* enrichment, marine bioprospecting, biotechnology

## Abstract

Deep-sea hydrothermal vents are amongst the most extreme environments on Earth and represent interesting targets for marine bioprospecting and biodiscovery. The microbial communities in hydrothermal vents are often dominated by chemolithoautotrophs utilizing simple chemical compounds, though the full extent of their heterotrophic abilities is still being explored. In the bioprocessing industry, where degradation of complex organic materials often is a major challenge, new microbial solutions are heavily needed. To meet these needs, we have developed novel *in situ* incubators and tested if deployment of recalcitrant materials from fish farming and wood-pulping industries introduced changes in the microbial community structure in hot marine hydrothermal sediments. The incubation chambers were deployed in sediments at the Bruse vent site located within the Jan Mayen vent field for 1 year, after which the microbial populations in the chambers were profiled by 16S rRNA Ion Torrent amplicon sequencing. A total of 921 operational taxonomic units (OTUs) were assigned into 74 different phyla where differences in community structure were observed depending on the incubated material, chamber depth below the sea floor and/or temperature. A high fraction of putative heterotrophic microbial lineages related to cultivated members within the Thermotogales were observed. However, considerable fractions of previously uncultivated and novel Thermotogales and Bacteroidetes were also identified. Moreover, several novel lineages (e.g., members within the DPANN superphylum, unidentified archaeal lineages, unclassified Thermoplasmatales and *Candidatus* division BRC-1 bacterium) of as-yet uncultivated thermophilic archaea and bacteria were identified. Overall, our data illustrate that amendment of hydrothermal vent communities by *in situ* incubation of biomass induces shifts in community structure toward increased fractions of heterotrophic microorganisms. The technologies utilized here could aid in subsequent metagenomics-based enzyme discovery for diverse industries.

## Introduction

With the growing demands for utilization of enzymes in industrial, medical and biotechnological applications, the search for new and specialized enzymes is of fundamental importance. Despite being well-known for hosting a high diversity of extremophiles with properties interesting for biotechnology, marine deep-sea hydrothermal vents remain under-sampled, limiting the use of this unique biodiversity in industry. In deep-sea hydrothermal vent ecosystems primary production is mainly based on chemosynthesis, however, hydrothermally altered biomass and/or deep ocean dissolved organic matter represent organic sources that may be utilized by heterotrophic microorganisms (Orcutt et al., 2011; Urich et al., 2013; Hawkes et al., 2015; Stokke et al., 2015; Meier et al., 2016; Steen et al., 2016; Dahle et al., 2018). Metagenome-assembled genomes have further revealed that diverse, heterotrophic microorganisms, with unknown physiologies, control the carbon flow in marine and hydrothermal deep-sea sediments via organic matter decomposition (Lloyd et al., 2013; Dombrowski et al., 2017). Hence, such microorganisms represent a great resource for finding new enzymes for biomedical, biotechnological, and industrial applications (Michalska et al., 2015).

With their ability to withstand the extreme environmental conditions found in these environments, hydrothermal vent microorganisms are highly recognized for their biotechnological potential. Hyperthermophiles are a valuable source for the industry of thermostable starch-processing, cellulose-degrading, xylan-degrading, chitin-degrading and proteolytic enzymes (Ferrer et al., 2007, 2009; Schmeisser et al., 2007; Shi et al., 2013; Fu et al., 2015; DeCastro et al., 2016; Wissuwa et al., 2016; Fredriksen et al., 2019). With the increasing demand for sustainable development based on the use of renewable raw materials, the importance of enzymes from hyperthermophiles in biomass conversions will likely increase.

Much of our current knowledge on the biotechnological application of (hyper) thermophiles is based on a few isolates obtained from hydrothermal vents affiliated with the bacterial order Thermotogales (*Thermotoga maritima*) and the archaeal order Thermococcales (*Pyrococcus furiosus*, *Thermococcus kodakarensis*) and on the archaeon *Sulfolobus sulfataricus* isolated from a Solfataric hot spring (Frock and Kelly, 2012). New reports of truly novel extreme thermophiles based on more than marginal differences in 16S rRNA phylogeny and subtle variations in growth physiology are infrequent (Frock and Kelly, 2012) despite the fact that molecular techniques have revealed a high diversity of yet uncultivated thermophilic bacteria and archaea in hot environments (Dombrowski et al., 2017, 2018).

In this study, we performed *in situ* amendment of deep-sea vent communities by deploying novel *in situ* incubators filled with industrially relevant polymeric biomass for 1 year in hydrothermal sediments at the Bruse vent site (Dahle et al., 2018; Stensland et al., 2019) on the Arctic Mid-Ocean Ridge (AMOR). Analysis of the resulting community structure by 16S rRNA gene amplicons revealed a high fraction of putative heterotrophic microbial lineages related to cultivated microorganisms. However, at the same time, the data revealed several novel lineages of as-yet uncultivated archaea and bacteria which provides a fundament for improved targeted metagenomics-based enzyme discovery as well as a starting point for discovery of novel microbial isolates.

## Materials and Methods

### Site Description and *in situ* Incubation

**FIGURE 1:**
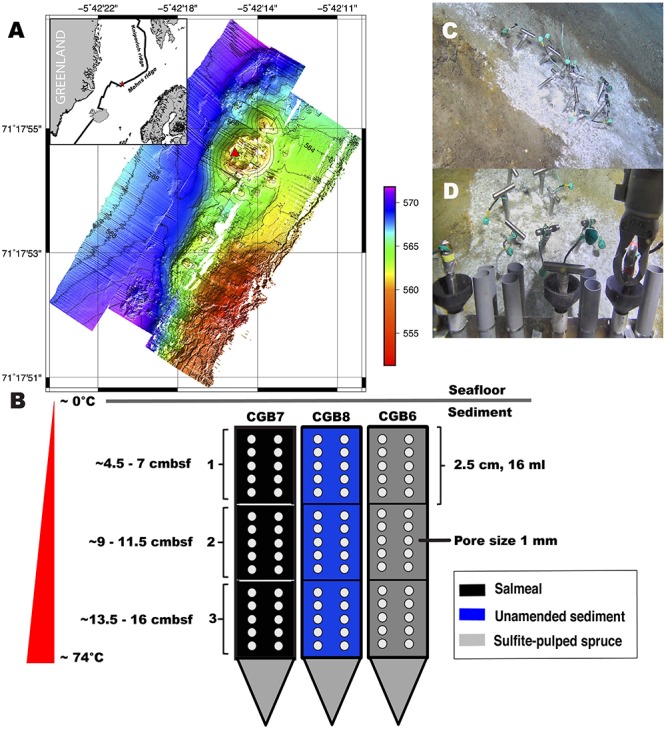
Sampling site and *in situ* incubation set-up. **(A)** Bathymetry map of the Bruse hydrothermal vent field with location (enclosed map). **(B)** *In situ* incubation set-up using Salmeal, Sulfite-pulped spruce and unamended sediment; cmbsf, centimeters below sea floor. Numbering, 1–3, relates to the different chambers given in Table 1. **(C)** incubators deployed in hot hydrothermal sediments at the Bruse vent field with white microbial mat on sediment surface and **(D)**, incubators being deployed from the ROV showing the gray incubator holsters mounted in a basket at the front of the ROV.

The Jan Mayen hydrothermal Vent Field (JMVF) is situated on the Arctic Mid-Ocean Ridge (AMOR) north of the Island Jan Mayen at 71°18′N and 5°47′W, about 50 km north of the Jan Mayen Fracture Zone (Pedersen et al., 2005; Schander et al., 2010). So far, three distinct venting areas/sites have been discovered in this field: the Troll Wall and Soria Moria vent sites (2005), and more recently, in 2014, the Bruse vent site, which is located approximately 5.5 km North-East of the Soria Moria vent site, at 71°18′N, 05°42′W and a depth of ∼570 meters (Dahle et al., 2018; Stensland et al., 2019). During a research cruise to the JMVF in July 2014, we deployed 3 titanium incubators each with 3 chambers (length 2.5 cm, volume 16 ml, per chamber) with 1 mm pores. The chambers were filled with ∼16 ml sediment sampled from the same vent site, supplemented with 1 g of industrial substrate selected within the biotech-project NorZymeD - enzyme development for Norwegian biomass^1^, as follows: CGB7, recalcitrant proteins/peptides from enzymatic hydrolysis of salmon bi-products referred to as Salmeal (Salmeal Active, Biomega Group AS, Norway). The Salmeal comprised >92% dry matter containing >68% protein, 11–17% lipid/fat, 10–15% minerals and 1.0–1.2% salt (NaCl); CGB6, unbleached Norway spruce (*Picea abies*) which had been subjected to a sulfite pulping pre-treatment method termed the BALI^TM^ process (Rødsrud et al., 2012; Sjöde et al., 2013) developed at Borregaard AS (Sarpsborg, Norway); CGB8, unammended sediment (control incubation). The spruce substrate used in CGB6 was dried at 40°C overnight and the particle size was reduced in a planetary ball mill PM 100 (Retsch, Haan, Germany) followed by sieving through a 0.85 mm screen. This substrate had a glucan content of 85%, while hemicelluloses (mannan and xylan) and acid insoluble lignin comprised 3% and 11%, respectively. Using three chambers allowed for incubation in a temperature gradient from approximately 74°C (samples labeled “3”) to 20°C (samples labeled “1”) (Figure 1B). The incubators were deployed using a remote operating vehicle (ROV) on board the research vessel G.O SARS. After 1 year of incubation, in July 2015, the incubators were recovered by the new ROV Ægir 6000 (NorMaR - Norwegian Marine Robotics Facility) and aliquots of each chambers were collected and stored at −20°C until processing.

### Geochemistry

Both high-temperature fluids (i.e., the near-pure Bruse endmember composition) and surrounding low-temperature diffuse fluids were collected using 160 mL titanium isobaric gas-tight fluid samplers (Seewald et al., 2002) deployed from the ROV Ægir 6000 during cruises with the G. O. Sars in 2016 and 2017, respectively. Maximum temperatures measured in real-time during sample collection for a given vent fluid are reported (uncertainty ±2°C). Low-temperature diffuse venting from the flanks of the main Bruse chimney mound (slightly deeper, but within a few meters of the incubators) were collected by inserting a ∼30 cm stainless steel borehole into the sediment (comprised mainly of anhydrite talus/sediment), in order to focus the fluids to the seafloor prior to collection by the snorkel of the fluid sampler. To prevent excessive seawater/sediment ingress into the borehole, it was crimped closed at its base, but perforated with drilled holes at a fixed distance (20–25 cm) from the sediment-water interface to allow hydrothermal venting to become focused and escape the sediment.

After recovery of the ROV, fluid samples were processed on board the ship within 12 h following protocols described in Reeves et al. (2011b). Briefly, aqueous H_2_ concentrations were determined onboard ship by molecular-sieve gas chromatography (GC) with thermal conductivity detection (TCD), following a syringe headspace extraction in N_2_ [uncertainties of ±10% (2 s)]. CH_4_ concentrations were determined by headspace gas chromatography onshore with fluid samples stored in HgCl_2_-poisoned serum vials (uncertainties of ±5% (2 s); following Reeves et al. (2011a). H_2_S was determined for high-temperature fluids by gravimetric precipitation as Ag_2_S, and for low-temperature fluids by electrochemical detection. For the latter, fluid samples were injected into a high-pH antioxidant buffer solution (SAOB, Thermo Fisher Scientific) and total dissolved H_2_S ([H_2_S] = [H_2_S] + [HS^–^] + [S^2–^]) subsequently measured as S^2–^ using a sulfide-specific (Ag^+^/S^2–^) electrode (e.g., after Pester et al., 2012). Fluid aliquots for determination of major elements (anions/cations) were transferred to acid-cleaned high-density polyethylene (HDPE) Nalgene bottles and analyzed onshore. These samples were sparged with N_2_ at sea to remove H_2_S prior to storage and analyzed either using ion chromatography (Cl, SO_4_) or inductively coupled plasma with optical emission spectroscopy (Mg, Na, K, Ca), with uncertainties for anions of ±5% (2 s) and for cations of ±2% (2 s).

### DNA Extraction, PCR and Ion Torrent 16S Amplicon Sequencing

DNA was extracted from 0.48–0.65 g of material from the individual chambers of the *in situ* incubators using the FastDNA spin kit for soil (MP Biomedicals, Irvine, CA, United States) according to the manufacturer’s protocol. The 16S rRNA gene amplicon libraries (V4–V5 regions) were prepared in a two-step PCR in order to minimize PCR biases introduced by the barcoded primers, as suggested by Berry et al. (2011). In the first PCR, samples were amplified using the universal primers 519f (5′- 5′-CAGCMGCCGCGGTAA-3′) (Ovreås et al., 1997) and 805r (5′-GACTACHVGGGTATCTAATCC) (Herlemann et al., 2011). The reactions contained 1× HotStarTaq master mix (Qiagen), 0.5 mM of each primer and sample DNA. The thermal program included 15 min activation of the Taq enzyme at 95°C, followed by 30 cycles of gene amplification, i.e., 30 s at 94°C, 30 s at 56°C and 90 s at 72°C. The final elongation was done at 72°C for 7 min. Triplicates of each sample/chamber were pooled, and then visualized and assessed by agarose gel electrophoresis. PCR products were purified using Agencourt AMPure XP beads (Beckman Coulter), with a 0.7 volume ratio between AMPure reagent and PCR product, according to the protocol supplied by Beckman Coulter. In the second PCR, the reactions contained 1x HotStarTaq master mix (Qiagen), 0.8 mM of each primer and approximately 100 ng template (quantified using Quantus Fluorometer, Promega Corporation), i.e., PCR product from the first reaction. The primers were barcoded and adapted to the sequencing technology used, where the forward primer includes an individual tag for sample identification. The thermal program was the same as before, however, only seven cycles of amplification were used. After purification with Agencourt AMPure XP beads, quantification using Quantus Fluorometer (Promega Corporation) and visualization using 1D gel electrophoresis, the nine samples were pooled in equimolar concentrations and the resulting solution was diluted to 40 pM (concentration measured spectrophotometrically prior to dilution based on the fluorochrome added to the purified PCR product). The 16S rRNA amplicon library was sequenced at the University of Bergen, Norway using Ion Torrent Personal Genome Machine (PGM) technology.

### Filtering, OTU Clustering and Taxonomic Classification

Sequences were filtered and clustered into operational taxonomic units (OTUs) using USEARCH (Edgar, 2010) and UPARSE (Edgar, 2013). Quality filtering and trimming to 250 bp was performed with the “-fastq_filter” command using options “-fastq_trunclen 250” and “-fastq_maxee 1”. Chimeric sequences were detected and removed with the “-uchime_ref” command using the Gold database as reference (available from “http://drive5.com/uchime/gold”). *De novo* OTU clustering was performed with a cut-off of 97% nucleotide sequence identity using the “cluster_otus” command. Taxonomic classification was performed in QIIME (Caporaso et al., 2010), using the command “summarize_taxa_through_plots.py” with the Silva version 128 as reference database^2^. A heatmap showing the relative abundance of different OTUs between the incubators were constructed using the “heatmap.2” function in the GPLOTS R package. A permutational ANOVA (PERMANOVA), as implemented in Vegan (Oksanen et al., 2018) with the “adonis2” function (Anderson, 2001), was used to test whether communities associated with different temperatures or substrate types were significantly different. The null hypothesis of the PERMANOVA test is that the metric centroid is the same in different sample groups (Anderson and Walsh, 2013). In order to test for differences in dispersion within groups of samples, with the null hypothesis that the average dispersion is the same within all sample groups (Anderson and Walsh, 2013), we used PERMDIST (Anderson, 2006) through the “betadisper” function in Vegan. Both “adonis2” and “betadisper” were run on a Bray-Curtis dissimilarity matrix generated from the OTU-table, using 999 permutations.

### Deposition of Sequence Data

Raw sequence data have been submitted to the Sequence Read Archive (SRA) under accession numbers SRX5783651- SRX5783659 as part of BioProject PRJNA296938.

## Results

### Chemical Compositions of Hydrothermal Fluids

The Bruse Vent Field, discovered in 2014, is located at 71°17.9′N, 05°42.2′W, about 5.5 km North-East from the Soria Moria Vent Field at a depth of ca. 560 m. Geochemical compositions of the high-temperature endmember fluids (242°C) venting through the hydrothermal chimneys, as well as the diffuse venting emanating from sediments where the incubators were deployed, are shown in Supplementary Table S1. The 2016 Bruse endmember composition is characterized by extremely high CH_4_ concentrations relative to other bare-rock hosted hydrothermal systems (German and Von Damm, 2003), but resembling levels observed in 2014 at Bruse (Dahle et al., 2018). Although maximum stable measured temperatures (240–242°C) are lower than the two-phase boundary for seawater at the seafloor (271°C), Cl contents lower than seawater indicate that the fluids have undergone phase separation, which may account for highly elevated CO_2_ and CH_4_ contents reported in Dahle et al. (2018), and high CH_4_ reported here. The very low H_2_ content and lack of significant NH_4_^+^ in the high-temperature fluid (Supplementary Table S1; Dahle et al., 2018; this study) do not indicate geochemical reactions of circulating hydrothermal fluids with crustal substrates other than mid-ocean ridge basalt, such as sediments, which would result in elevated NH_4_^+^ and H_2_ (e.g., Lilley et al., 1993). Instead, the fluids resemble fluids from other basalt-hosted hydrothermal systems on the Mid-Atlantic Ridge that are characterized by phase separation at relatively low seafloor pressures (depths), such as Menez Gwen (Reeves et al., 2011b).

Low-temperature venting from the surrounding mound sediment is evidently derived from the Bruse endmember fluid. In the low-temperature samples, the elements K (Supplementary Figure S1) and Na (Supplementary Table S1, not plotted) plot on simple mixing lines with their respective endmember concentrations, implying the sampled diffuse fluid formed by dilution of the endmember composition with subsurface seawater, since these elements generally behave conservatively during subsurface mixing (Reeves et al., 2011a). In contrast, Ca and SO_4_ display deviations from simple mixing behavior (enrichments) that point to dissolution of the anhydrite sediment through which the fluids emanate, which was composed of fine needle-like anhydrite crystals likely derived from chimney debris. Anhydrite is typically insoluble in seawater-like solutions above temperatures of ∼150°C (Bischoff and Dickson, 1975), but soluble below this, and has been observed to dissolve in other diffuse hydrothermal vents characterized by anhydrite sediment substrate (Reeves et al., 2011a).

### Experimental Design and Amplicon Sequencing

Novel perforated titanium incubators were constructed in order to perform *in situ* microbial enrichments in deep-sea hydrothermal sediments. These incubators were vertically separated into three chambers, which allowed assessing multiple positions along the temperature gradient (Figure 1B). In 2014, three incubators were deployed in a hydrothermal sediment at the Bruse vent field situated within the Jan Mayen Hydrothermal Vent Fields (JMHVFs) (Figure 1A). After 1 year of incubation the incubators were collected (2015) and sampled for microbial composition. At deployment in 2014, the *in situ* sediment temperature was measured with an external temperature probe attached to the ROV to ∼74°C at 20 cm sediment depth. An *in situ* temperature profile logged from 2016 to 2017 (data not shown) indicated that the sediment temperature at the site was stable, meaning that it is safe to assume that the temperature during the 2014–2015 incubation also was stable. Sequencing of DNA retrieved from the nine incubator chambers yielded 547 349 16S rRNA amplicon reads in total. Table 1 and Supplementary Table S2 provide an overview of the samples and the sequencing statistics.

**TABLE 1:** Sample and sequencing overview for the *in situ* enrichments.

**Sample name**	**Biosample**	**Short name**	**Substrate**	**Sediment depth**	**Temperature**(°C)	**Filtered amplicons**
CGB12_MID81_IS7_1	SAMN06885931	CGB7_1	Salmeal	∼4.5–7 cm	∼30	33232
CGB12_MID82_IS7_2	SAMN06885933	CGB7_2		∼9–11 cm	∼50	20136
CGB12_MID83_IS7_3	SAMN09901157	CGB7_3		∼13.5–16 cm	∼75	29967
CGB11_MID92_IS8_1	SAMN09901172	CGB8_1	Unamended sediment	∼4.5–7 cm	∼30	44308
CGB11_MID93_IS8_2	SAMN09901206	CGB8_2		∼9–11 cm	∼50	39474
CGB11_MID94_IS8_3	SAMN09901209	CGB8_3		∼13.5–16 cm	∼75	32820
CGB11_MID89_IS6_1	SAMN09901170	CGB6_1	Sulfite-pulped spruce	∼4.5–7 cm	∼30	33303
CGB11_MID90_IS6_2	SAMN09768205	CGB6_2		∼9–11 cm	∼50	26849
CGB11_MID91_IS6_3	SAMN09768207	CGB6_3		∼13.5–16 cm	∼75	35463

**FIGURE 2:**
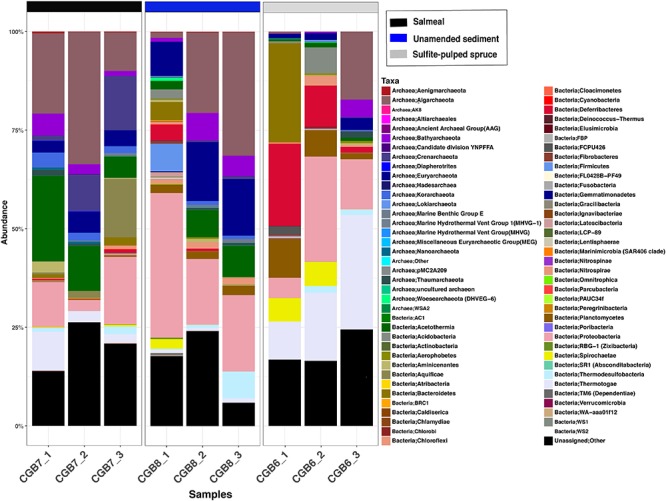
Relative taxonomic abundances of *in situ* enrichments at the phylum level. The sample codes are explained in Figure 1 and Table 1.

**FIGURE 3:**
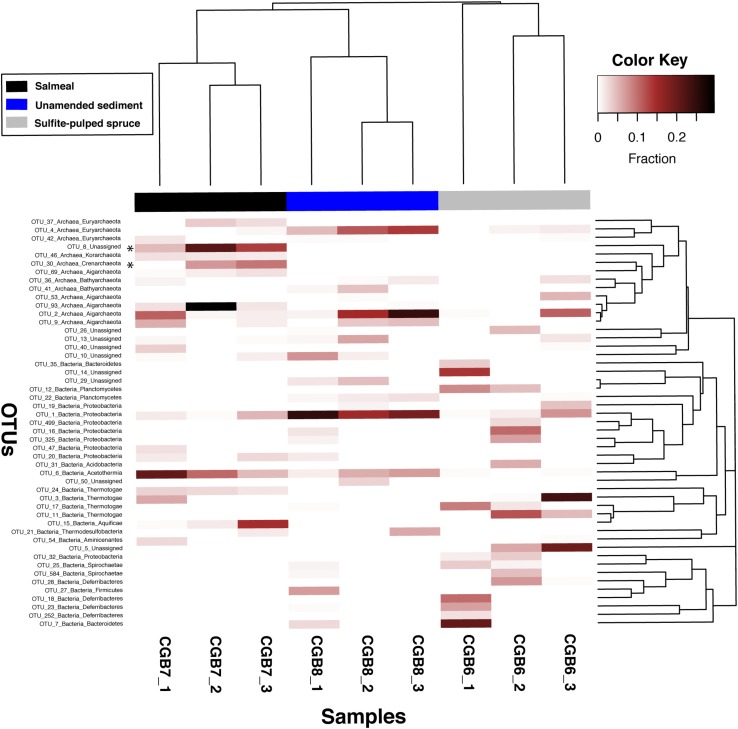
Heatmap showing the distribution of the 50 most abundant OTUs > 2% relative abundance within each of the collected *in situ* chambers. Samples are clustered by complete hierarchical clustering (top) and OTUs are clustered phylogenetically (right) using the neighbor-joining algorithm implemented in FastTree (Price et al., 2010).

The results of the community analyses are provided in Figure 2, showing the relative taxonomic abundances of *in situ* enrichments at the phylum level, Figure 3, with a heatmap showing the distribution of the 50 most abundant OTUs >2% relative abundance within each of the collected *in situ* chambers, and Supplementary Tables S3–S5, showing the most abundant OTUs (>2% relative abundance) of the three incubators with corresponding SILVA taxonomic assignment. In addition, Supplementary Figure S2 show the combined bacterial and archaeal 16S rRNA phylogeny using all identified OTUs.

### Microbial Community Structure of Unamended Sediment (CGB8_1-3)

The unamended sediment control samples (CGB8_1-3) showed an increasing fraction of Archaea as temperature increased with sediment depth in the three chambers, varying from 12.3% in the upper chamber to 54.1% in the lower chamber (see Figure 2 and Supplementary Table S3 for data concerning CGB8_1-3). Members within Aigarchaeota and Euryarchaeota dominated the deepest chamber, representing 31.3 and 14.3% of the total amplicons, respectively. OTU2 was the most dominant of the Aigarchaeota amplicons, representing 24.5% of the total community, OTU9 comprised 4.6% and OTU36 with 1.7% of the community. The Aigarchaeota amplicons within OTU2 showed 100% similarity to an uncultured archaeon from the Iheya North hydrothermal field, Okinawa Trough (Supplementary Table S3). Within Euryarchaeota, 13.8% of the amplicons grouped into OTU4 with 96% similarity to ANME-1a from a hydrothermal sediment at Guayamas Basin (Lever and Teske, 2015). In the upper chamber (CGB8_1), at lower temperature, the proteobacteria dominated at the phylum level (38.8%), with OTU1 representing the most abundant group (25%) (Figure 3). OTU1 showed 97% identity to an uncultured deltaproteobacterium from a hydrothermally influenced sediment at Guayamas Basin (Holler et al., 2011) and *Desulfofervidus auxii*, a hydrogenotrophic sulfate-reducing bacterium known to be involved in thermophilic anaerobic oxidation of methane (Krukenberg et al., 2016). OTU1 was also the most dominant amongst the Proteobacteria in the high temperature CGB8_3 chamber, representing 18.8% of the amplicons. In the deepest chamber, only 6.1% remained unassigned at the phylum level, whereas for CGB8_2 and CGB8_1 24.1 and 18.9% of the amplicons remained unassigned at the phylum level, respectively.

### *In situ* Enrichment With BALI^TM^ Pre-treated Spruce (CGB6_1-3)

In the BALI^TM^ pre-treated spruce incubator, the bacterial population dominated all three chambers (81.2 – 48.8%; Figure 2; Supplementary Table S4. In the upper chamber (CGB6_1), Bacteroidetes dominated the bacterial community (25.2%) with OTU7 (20.5%) and OTU35 (3.5%) as the dominating sequence types (Figure 3). OTU7 showed 99% identity to an uncultured bacterium from sulfide deposits at the Southern Mariana Trough. The upper chamber also showed a high fraction of Deferribacteres (20.8%) with OTU18 (10.2%), OTU23 (6.6%) and OTU252 (2.3%) as the predominant sequence types. OTU18 showed 99% identity to an uncultured bacterium from methane- and short chain alkane-rich hydrothermal sediments of Guaymas Basin, while OTU23 and OTU252 showed 100 and 96% identity, respectively, to uncultured bacteria from the rhizosphere of *Haloxylon recurvum* (plant family Amaranthaceae) under highly saline conditions. Searches in the NCBI refseq database showed that all three OTUs had top hits against a Candidate division KSB1 bacterium with 91.5, 95.1, and 93.0% identity, respectively. The upper chamber also showed relatively large fractions of Planctomycetes (10%), Thermotogae (9.6%), Spirochaetae (5.9%) and Proteobacteria (5.1%) (Figure 2).

In the middle chamber (CGB6_2), Proteobacteria and Thermotogae dominated the microbial community with 26.7% and 17.2%, respectively. Furthermore, Deferribacteres (10.5%), Acidobacteria (6.6%), Planctomycetes (6.7%), Spirochaetae (6.1%) and Chlororflexi (2.5%) comprised larger fractions (Figure 2 and Supplementary Table S4). OTUs within Thermotogae comprised significant fractions in the CGB6 incubator at all depths (Figure 2, Table 2, and Supplementary Table S4). In the deepest chamber (CGB6_3) Thermotogae dominated the microbial community with 29% of the amplicons with OTU3 representing 23.5% of the community and showed 99% similarity to uncultured Thermotoga from alkaline hot springs of Ambitle Island, Papua New Guinea (Supplementary Table S4). However, OTU3 showed only 88.4% to the 16S rRNA gene to the closest cultivated strain within the Thermotogales, *Thermosipho melanesiensis* strain 433. In the upper chamber, OTU17 represented the dominating OTU with 9.14% of the community and showed highest identity to an uncultured bacterium (94%) from hydrothermal sediments, Southern Okinawa trough (Supplementary Table S4). In the mid-chamber, OTU11 comprised 12% of the microbial community and showed 94% identity to a Thermotogae from a high temperature reservoir enrichment culture (Supplementary Table S4).

**TABLE 2:** Fraction (%) of Thermotogae OTUs in the incubators.

	**Salmeal**			**Unamended sediment**			**Sulfite-pulped spruce**		
**Thermotogae OTUs**	**CGB7_1**	**CGB7_2**	**CGB7_3**	**CGB8_1**	**CGB8_2**	**CGB8_3**	**CGB6_1**	**CGB6_2**	**CGB6_3**
OTU_11	0.02	0.01	0.02	0.17	0.09	0.00	0.04	11.98	4.85
OTU_17	0.00	0.00	0.00	0.41	0.00	0.00	9.14	2.06	0.00
OTU_24	2.97	2.82	2.06	0.01	0.14	0.17	0.00	0.00	0.18
OTU_3	5.99	0.01	0.07	0.00	0.06	0.10	0.00	0.35	23.55

Compared to CGB7 (Salmeal), OTU17 were unique to the BALI^TM^ pre-treated spruce incubator with only a low fraction in the upper chamber of the unamended sediment (CGB8_1). Table 2 summarizes the fractions of Thermotogae OTUs in all three incubators.

The archaeal population comprised a low fraction of the microbial community in the two uppermost chambers (1.9 and 2.7%), however, increased to 26.9% in the deepest chamber (CGB6_3) with Aigarchaeota representing 17.2%, Bathyarchaeota 4.5%, Euryarchaeota 3.1% and Thaumarchaeota 1.6% of the community. Similar to the deepest chamber with unamended sediment (CGB8_3), the Aigarchaeota sequences were dominated by OTU2 (11.1%). In addition, 5.1% of the Aigarchaeota in CGB6_3 were affiliated with OTU53. The unassigned fraction of the microbial community increased with chamber depth and temperature (16.8–24.5%) and was dominated in CGB6_1 by OTU14 (14.1%), in CGB6_2 by OTU26 (4.9%) and OTU5 (6.1%), and in CGB6_3 by OTU5 (19.5%). These OTUs were unique to the BALI^TM^ pre-treated spruce incubator. OTU14 showed the highest identity (91%) to an uncultured bacterium from a deep-sea mud volcano from the East Mediterranean Sea, OTU26 to an uncultured archaeon from a Sulfidic monimolimnion of Ace Lake, Antarctica (91%), and OTU5 to an uncultured bacterium (88%) from sediments of an eutrophic shallow lake (Supplementary Table S4). The 16S rRNA phylogeny (Supplementary Figure S2) placed OTU14 and OTU26 amongst the *Candidatus* division BRC-1 and Woesearchaeota within the superphylum DPANN, respectively.

### *In situ* Enrichment With Recalcitrant Proteins (Salmeal; CGB7_1-3)

In the incubator with recalcitrant proteins (Salmeal), the bacterial population dominated the upper and deepest chamber with 49.4 and 47.1% of the microbial community, while the archaeal population dominated the middle chamber (54.2%) (Figure 2 and Supplementary Table S5). Similar to the unamended sediment sample, the Aigarchaeota dominated the archaeal fraction (33.5%) with OTU2 as the predominant sequence type in the upper chamber (11.1%) (Figure 3). In the middle chamber, with the largest archaeal fraction, OTU93 within Aigarchaeota, represented 29.5% of the total community. Clustering within Archaea (Figure 3) and unique to the Salmeal sample, the unassigned OTU8 represented 21.4% of the community in the middle chamber, having the highest identity to an uncultured euryarchaeote from a hydrothermal chimney at Lucky Strike (88%). Intriguingly, the 16S rRNA phylogeny (Supplementary Figure S2) placed this OTU amongst the archaeal phylum Diapherotrites within the superphylum DPANN.

Significant fractions of Acetothermia were observed in the CGB7 incubator (21.7–5.3%), with OTU6 as the predominant sequence type (20.5%–4.8%), showing 99% identity to an uncultured bacterium from a shallow hydrothermal vent field from the Mexican Pacific West coast (Supplementary Table S4). OTU6 also showed 92% identity to the 16S rRNA gene of the published genome of the *Candidatus* Acetothermia bacterium Ran1 recovered using anaerobic digesters from wastewater treatment plants (Hao et al., 2018).

A larger fraction of Proteobacteria was observed in the upper (CGB7_1) and deepest (CGB7_3) chamber (11.4 and 17.1%), whereas this fraction was only 2.7% in the middle chamber. Similar to the BALI sample (CGB6), the Proteobacteria community was dispersed within several OTUs, with OTU1 and OTU20 as most abundant in the deepest chamber, with 5.3 and 2.8%, respectively, while OTU47 represented 2.1% of the microbial community in the upper chamber. Intriguingly, an increasing fraction of Crenarchaeota was observed with depth and temperature (1.3–13.7%, Figure 2) in the CGB7 incubator, of which 11.1% was classified to the Desulfurococcaceae in the deepest chamber. Unique to the Salmeal sample, OTU30 represented the dominating sequence type (Figure 3) with 9.6% of the microbial community in the lower chamber, showing 99% identity to an Uncultured Desulfurococcales archaeon, HTM866S-A1 (Supplementary Table S5), and 96% 16S rRNA identity to the cultivated *Thermogladius cellulolyticus*. *T. cellulolyticus* was isolated from a hot spring in the Uzon Caldera in Kamchatka (Mardanov et al., 2012) and ferments various cellulose substrates (filter paper, microcrystalline and carboxymethyl cellulose).

In addition to the high fraction of Crenarchaeota, a unique feature of the Salmeal sample was the increasing fraction of Aquificae with depth and temperature (0.5–14.7%) with OTU15 as the dominating sequence type (0.47 to 14.8%). Except from a low presence in the deepest chamber of the unamended sediment (CGB8_3, 0.01%), OTU15 was unique to the Salmeal incubator showing 97% identity to the obligate chemolitoautotroph *Thermosulfidibacter takaii* isolated from a deep-sea hydrothermal field, Southern Okinawa Trough (Nunoura et al., 2008; Supplementary Table S5).

The salmeal incubator also contained elevated OTU fractions (OTU37;Top chamber and OTU42;mid-and bottom chamber) related to uncultivated members of the Thermoplasmatales (Supplementary Figure S2).

## Discussion

Our study was designed to improve the potential for biodiscovery of novel enzymes for use in biorefining and bioconversion of industrially relevant substrates. To improve the chances for collecting larger amounts of more relevant microbial biomass, we developed deep-sea *in situ* incubators, which were used for targeted enrichment of (hyper)thermophilic organotrophic microorganisms using substrates from the wood pulp industry (CGB6) and recalcitrant rest materials from fish farming (CGB7). The potential for enzyme discovery from these enrichments has recently been shown by the metagenome-based discovery of three novel enzymes, a thermostable alginate lyase belonging to the Polysaccharide Lyase family 7 (Vuoristo et al., 2019), a thermostable GH10 xylanase with broad substrate specificity (Fredriksen et al., 2019) and an exceptionally stable GH9 cellulase with an apparent melting temperature of 105°C (Stepnov et al., 2019). Overall, the data show clear shifts in the community structure for both *in situ* enrichments relative to the unamended control sediment chambers as well as between the two different enrichments (Figures 2, 3). Moreover, and expectedly considering the large temperature gradient, shifts were also observed between chambers containing the same substrate, being most pronounced for the amended samples, especially the one with *BALI* pre-treated spruce (Supplementary Figure S3). Furthermore, a permutational analysis of variance (PERMANOVA) showed that samples formed significantly distinct groups according to substrate (*F* = 2.5, *p* = 0.007). Using PERMDIST, we found no evidence for differences in dispersion within these sample groups (*F* = 1.5, *p* = 0.3).

The hydrothermal signature of the sediment fluids was reflected in the microbial populations within the incubator containing unamended sediment (CGB8_1-3). High methane and sulfate supported the growth of anaerobic methane oxidizers and sulfate reducing bacteria as well as the high abundance of Aigarchaeota (OTU2). A high fraction of Aigarchaeota was also detected in the deepest chamber of CGB6. Interestingly, the abundant Aigarchaeota OTU was different (OTU93) than the unamended sediment suggesting a heterotrophic lifestyle. Although most described metagenomic bins of Aigarchaeota suggests a chemolitotrophic lifestyle (Hua et al., 2018), some are described as aerobic, chemoorganoheterophs with autotrophic potential, such as the filamentous aigarchaeon *Candidatus* ‘Calditenuis aerorheumensis (Beam et al., 2015). The identification of putative cellulases from metagenomic Aigarchaeota bins (Hua et al., 2018) further suggests the capacity to degrade complex carbohydrates and cycling of organic matter in deep-sea sediments which fits well with the cellulose containing substrate used in the CGB6 incubator.

In the BALI^TM^ pre-treated spruce enrichments (CGB6_1-3) we detected high abundances of putative organotrophs, with an increasing fraction of Thermotogales with temperature, and of Bacteroidetes at moderate temperatures (upper chamber, CGB6_1). Although members of the Thermotogales are well known for degrading cellulose (Ruttersmith and Daniel, 1991; Bronnenmeier et al., 1995; Bok et al., 1998; Wery et al., 2001; Ben Hania et al., 2012) incubation with a specific substrate within the wood pulp industry resulted in enrichment of novel sequence types within Thermotogales with low sequence identity to known cultured strains. Interestingly, different OTUs are dominating the three chambers suggesting a differentiation due to increased temperature.

In addition to a high fraction of Bacteroidetes at moderate temperatures (CGB6_1, upper chamber, 25.2%), the high fraction of Deferribacteres in the upper- and middle chamber (20.8 and 10.5%, respectively) is consistent with previous observations from deep-sea hydrothermal systems where Bacteroidetes and chemoorganotrophic Deferribacteres genomic bins comprised abundant transcript fractions for glycoside hydrolases (GHs), and thus suggested to be important players in organic matter utilization/breakdown in deep-sea hydrothermal vents (Urich et al., 2013; Stokke et al., 2015; Li et al., 2016).

Recent studies from marine and terrestrial subsurface environments has identified a variety of extracellular enzymes adapted to degrade detrital sedimentary organic matter (Michalska et al., 2015; Steen et al., 2019). Interestingly, the Salmeal sample (CGB7) contained a high fraction of unique unassigned Archaea (OTU8) as well as high abundance of uncultivated Acetothermia (OTU6). Genomic reconstruction of an Acetothermia bacterium from anaerobic digesters has suggested that this species has an anaerobic chemoorganotrophic lifestyle, fermenting peptides, amino acids and simple sugars to acetate, formate and hydrogen (Hao et al., 2018), which fits well with the high content of recalcitrant protein/peptides in the Salmeal substrate (>68%). Although classified as unassigned, OTU8 grouped within the Diapherotrites in the DPANN superphylum (Supplementary Figure S2). DPANN represent a large group of archaea characterized by reduced genome sizes and limited metabolic capabilities suggesting a host-associated lifestyle (Golyshina et al., 2017; Dombrowski et al., 2019). However, analysis of single amplified genomes of members within the Diapherotrites point to the possibility of a free-living lifestyle using a narrow range of substrates such as ribose, polyhydroxybutyrate and several amino acids to acetyl-coenzyme A (Youssef et al., 2015). Based on the substrate used in the CGB7 incubation, with high protein/peptide content, it is possible that OTU8 represents a novel free-living DPANN.

An interesting observation in the deepest Salmeal chamber (CGB7_3) is the high fraction of Aquificae represented by a single OTU (OTU15, 14.7%). Members within the Aquificae represents (hyper)thermophilic microorganisms inhabiting marine and terrestrial hydrothermal systems globally (Reysenbach et al., 2002; Ferrera et al., 2007) and important primary producers in terrestrial (Reysenbach et al., 1994; Spear et al., 2005) and deep-sea hydrothermal systems (Mino et al., 2013). They are described as capable of both chemolithotrophy (Ferrera et al., 2007; Nunoura et al., 2008) and heterotrophy (Caldwell et al., 2010). Although OTU15 share 97% 16S rRNA identity to the obligate chemolitoautotroph *Thermosulfidibacter takaii* (Nunoura et al., 2008) the high abundance suggest the capability of heterotrophy in the OTU15 sequence type.

Hence, the current study shows that our *in situ* enrichment strategy induced major shifts in the composition of deep-sea microbial communities, and favored the enrichment of uncultivated and novel heterotrophic microbial lineages (Figures 2, 3 and Supplementary Figure S2). These novel lineages, which are likely adapted to dealing with the industrial substrates in the incubation chambers, provide novel sources for enzyme mining and metagenomic data derived from the enriched microbial biomass has already been successfully exploited in enzyme discovery. Furthermore, this study sets the baseline for genome-centric exploration of the genetic variety and metabolic versatility of as-yet uncultured archaeal and bacterial lineages from the Arctic deep-sea hydrothermal vent fields.

## Data Availability Statement

The datasets generated for this study can be found in the Raw sequence data have been submitted to the Sequence Read Archive (SRA) under accession numbers SRX5783651–SRX5783659 as part of BioProject PRJNA296938.

## Author Contributions

RS, VE, and IS conceived and designed the experiments. A-EF extracted DNA and obtained the 16S rRNA gene amplicons. HD performed the bioinformatic analysis on 16S rRNA reads. ER, TV, and FV conducted the geochemical sampling and analysis. SL made the map of bathymetric data from the Bruse vent field. RP was research cruise leader and chose sampling site. RS and IS analyzed the data and wrote the manuscript. All coauthors edited the manuscript.

## Conflict of Interest

The authors declare that the research was conducted in the absence of any commercial or financial relationships that could be construed as a potential conflict of interest.

## Footnotes

1 https://norzymed.nmbu.no/

2 www.arb-silva.de

## Supplementary Material

FIGURE S1 Plots of selected species CH_4_ **(A)**, SO_4_ **(B)**, Ca **(C)**, and K **(D)** versus Mg for the Bruse high-temperature (242°C) fluid and associated nearby diffuse venting (<54°C). *Y*-axis arrows denote endmember compositions of Bruse high-temperature fluid (229°C) reported in Dahle et al. (2018) for comparison. In the 2016–2017 data, K displays relatively conservative dilution behavior between the endmember and bottom seawater (BSW), clearly supporting the Bruse endmember as the most reasonable source fluid for the diffuse vent fluids emanating from within the same mound. With higher concentrations than what is possible by simple mixing with seawater, Ca and SO_4_, in contrast display non-conservative behavior, which is most likely due to dissolution of the anhydrite sediment comprising the Bruse mound.

FIGURE S2 A combined bacterial and archaeal 16S rRNA phylogeny was constructed using all OTUs and neighboring sequences (black branch nodes) as identified using the Sina aligner v.1.2.11 (Pruesse et al., 2012). All sequences (3158) were aligned with MAFFT-LINSi (Katoh et al., 2002) and trimmed with trimal (224 bp) (Capella-Gutiérrez et al., 2009). Tree construction was performed using FastTree (Price et al., 2010) and visualized in iTOL (Letunic and Bork, 2016). OTUs with blue text labels and branch nodes are those represented in the total dataset with above 2% abundance, while branch nodes in red are OTUs below 2% abundance.

FIGURE S3 PCoA plot of *in situ* incubator samples showing inter- and intra-distances related to substrate and depth/temperature. The plot was made using Qiime (Caporaso et al., 2010).

TABLE S1 Table of measured concentrations of hydrothermal fluid samples from the Bruse high-temperature vent, and associated diffuse venting sediment. A zero-Mg endmember composition is calculated for the high temperature fluid samples, whose Mg concentrations approach that of zero, with only negligible contributions from sampler dead volume seawater (Reeves et al., 2011a). Calculation of a zero-Mg endmember is not meaningful for the highly diluted and cooled associated diffuse vent fluids.

TABLE S2 Sequencing statistics and 16S rRNA read filtering.

TABLE S3 Most abundant OTUs in the unamended sediment samples. Data are shown for each chamber with increasing sediment depth and temperature (CGB8_1, CGB8_2, and CGB8_3) as relative abundance, with corresponding SILVA taxonomic assignment, closest blast hit and environment in the NCBI non-redundant database.

TABLE S4 Most abundant OTUs in BALI^TM^ pre-treated spruce sample. Data are shown for each chamber with increasing sediment depth and temperature (CGB6_1, CGB6_2, and CGB6_3) as relative abundance, with corresponding SILVA taxonomic assignment, closest blast hit and environment in the NCBI non-redundant database.

TABLE S5 Most abundant OTUs in the Salmeal sample. Data are shown for each chamber with increasing sediment depth and temperature (CGB7_1, CGB7_2, and CGB7_3) as relative abundance, with corresponding SILVA taxonomic assignment, closest blast hit and environment in the NCBI non-redundant database.
