# Supplementary figures and images for: Tailoring Hydrothermal Vent Biodiversity Toward Improved Biodiscovery Using a Novel in situ Enrichment Strategy
Source: Front Microbiol. 2020 Feb 21;11:249. doi: 10.3389/fmicb.2020.00249 (PMC7046548; doi:10.3389/fmicb.2020.00249)

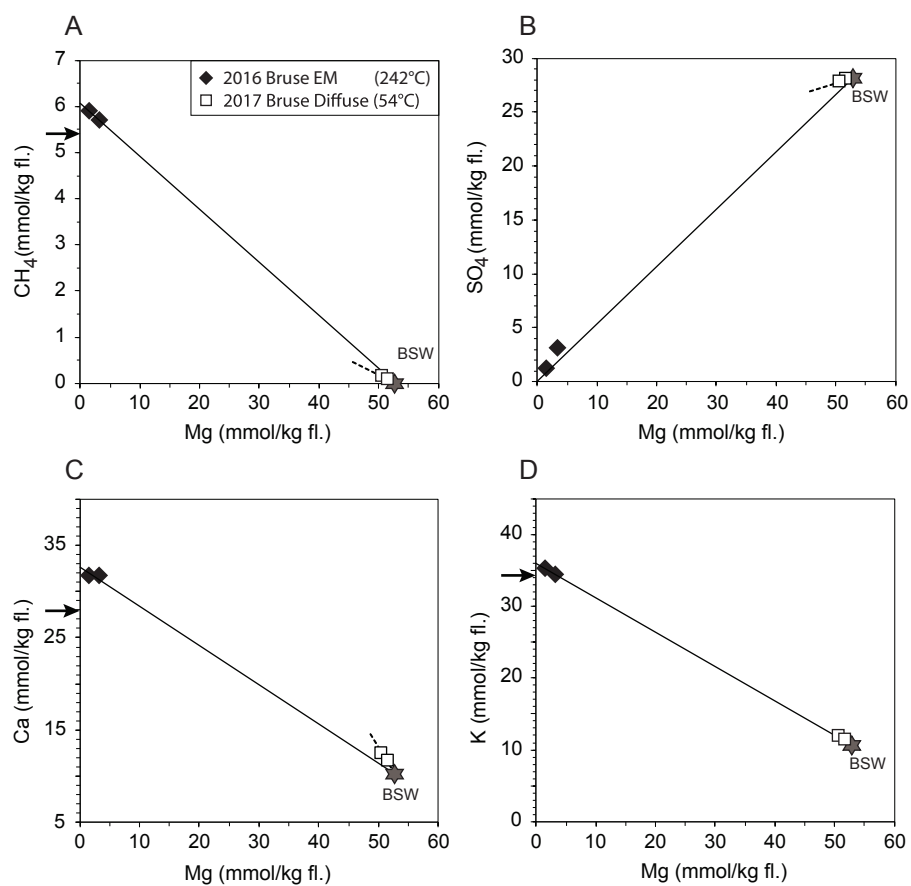

Supplementary Figure S1

Supplement: FIGURE S1 — Plots of selected species CH4 (A), SO4 (B), Ca (C), and K (D) versus Mg for the Bruse high-temperature (242°C) fluid and associated nearby diffuse venting (<54°C). Y-axis arrows denote endmember compositions of Bruse high-temperature fluid (229°C) reported in Dahle et al. (2018) for comparison. In the 2016–2017 data, K displays relatively conservative dilution behavior between the endmember and bottom seawater (BSW), clearly supporting the Bruse endmember as the most reasonable source fluid for the diffuse vent fluids emanating from within the same mound. With higher concentrations than what is possible by simple mixing with seawater, Ca and SO4, in contrast display non-conservative behavior, which is most likely due to dissolution of the anhydrite sediment comprising the Bruse mound. [file Image_1.pdf]

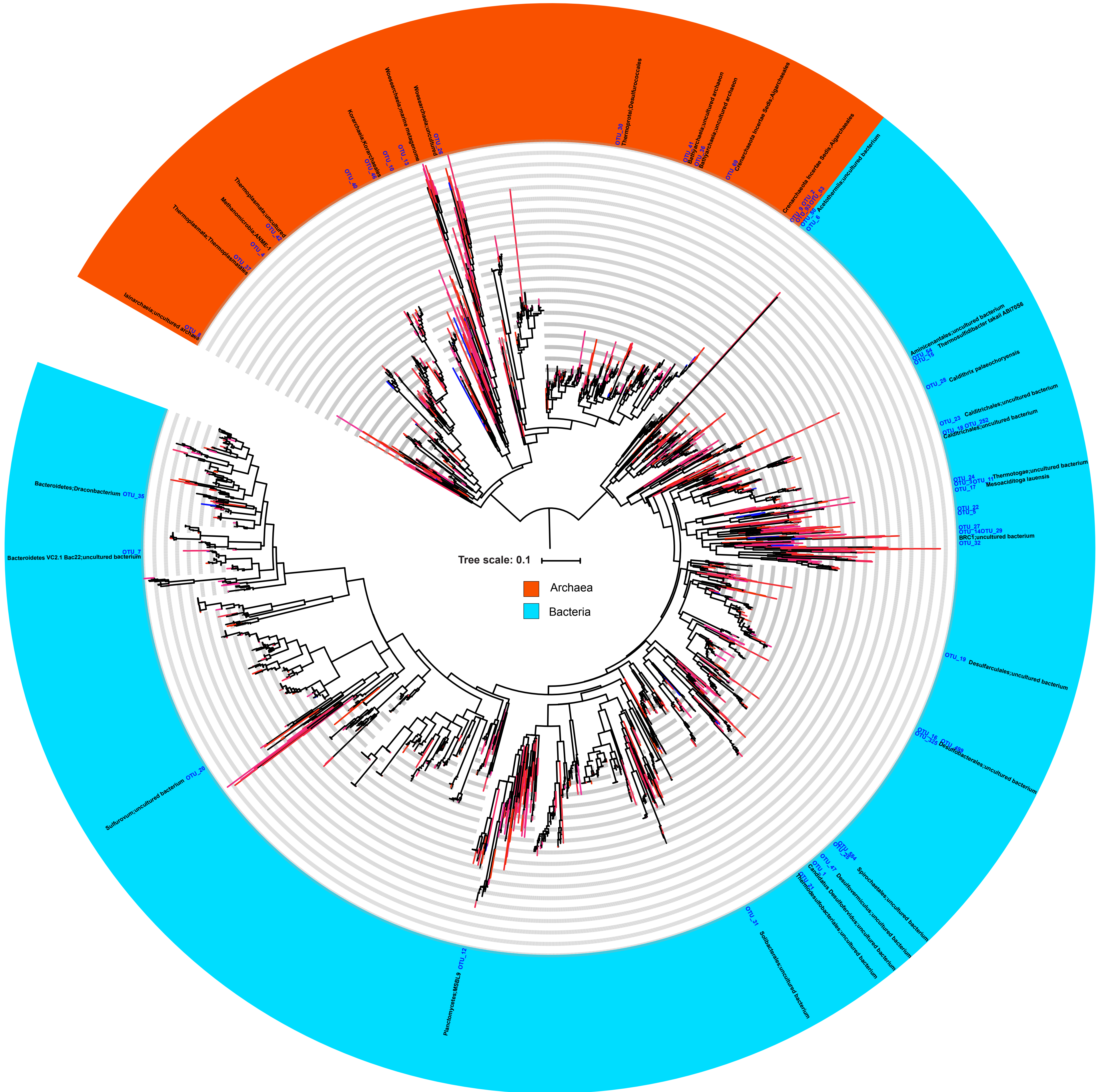

Supplementary Figure S2

Supplement: FIGURE S2 — A combined bacterial and archaeal 16S rRNA phylogeny was constructed using all OTUs and neighboring sequences (black branch nodes) as identified using the Sina aligner v.1.2.11 (Pruesse et al., 2012). All sequences (3158) were aligned with MAFFT-LINSi (Katoh et al., 2002) and trimmed with trimal (224 bp) (Capella-Gutiérrez et al., 2009). Tree construction was performed using FastTree (Price et al., 2010) and visualized in iTOL (Letunic and Bork, 2016). OTUs with blue text labels and branch nodes are those represented in the total dataset with above 2% abundance, while branch nodes in red are OTUs below 2% abundance. [file Image_2.pdf]

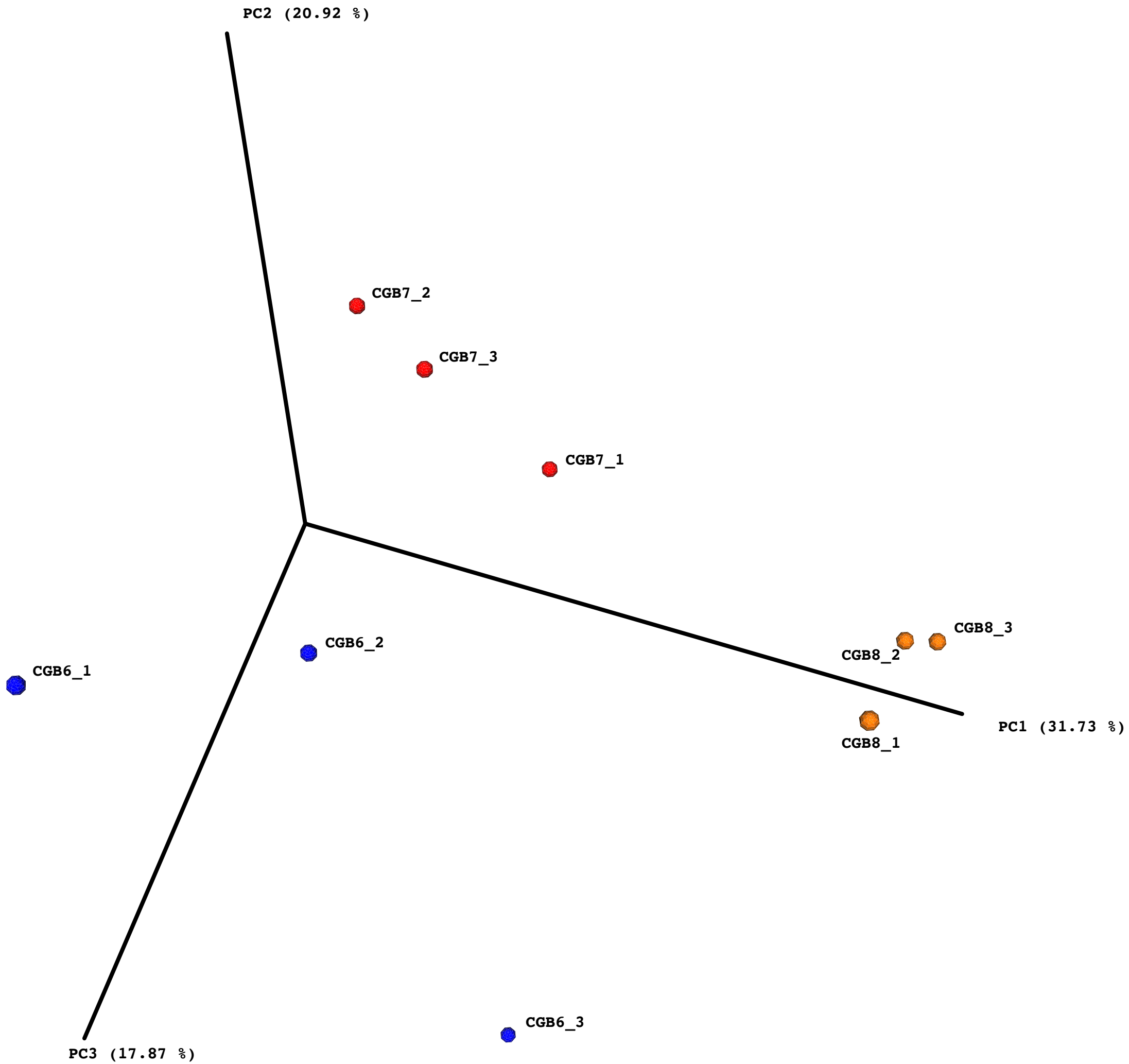

Supplementary Figure S3

Supplement: FIGURE S3 — PCoA plot of in situ incubator samples showing inter- and intra-distances related to substrate and depth/temperature. The plot was made using Qiime (Caporaso et al., 2010). [file Image_3.pdf]
