# Supplementary material for: Tailoring Hydrothermal Vent Biodiversity Toward Improved Biodiscovery Using a Novel in situ Enrichment Strategy
Source: Front Microbiol. 2020 Feb 21;11:249. doi: 10.3389/fmicb.2020.00249 (PMC7046548; doi:10.3389/fmicb.2020.00249)
Supplement: TABLE S1 — Table of measured concentrations of hydrothermal fluid samples from the Bruse high-temperature vent, and associated diffuse venting sediment. A zero-Mg endmember composition is calculated for the high temperature fluid samples, whose Mg concentrations approach that of zero, with only negligible contributions from sampler dead volume seawater (Reeves et al., 2011a). Calculation of a zero-Mg endmember is not meaningful for the highly diluted and cooled associated diffuse vent fluids. [file Table_1.docx]

**Table S1.** Table of measured concentrations of hydrothermal fluid samples from the Bruse high-temperature vent, and associated diffuse venting sediment. A zero-Mg endmember composition is calculated for the high temperature fluid samples, whose Mg concentrations approach that of zero, with only negligible contributions from sampler dead volume seawater (Reeves et al. 2011a). Calculation of a zero-Mg endmember is not meaningful for the highly diluted and cooled associated diffuse vent fluids.

|  | **Depth** | **T_max_** | **pH** | **Mg** | **H_2_** | **H_2_S** | **CH_4_** | **Na** | **K** | **Ca** | **Cl** | **SO_4_** |
| --- | --- | --- | --- | --- | --- | --- | --- | --- | --- | --- | --- | --- |
| **Sample** | **m** | **°C** | **(25°C)** | **mm** | **µM** | **mM** | **mm** | **mm** | **mm** | **mm** | **mm** | **mm** |
| **Bruse High-Temperature Vent (71°17.910`N, 5°42.240`W)** | | | | |  |  |  |  |  |  |  |  |
| GS16B-16-ROV05-IGT2 | 561 | 240 | 4.9 | 3.4 | 16 | 1.0 | 5.7 | 414 | 34.2 | 31.7 | - | 3.1 |
| GS16B-16-ROV05-IGT3 | 561 | 242 | 4.8 | 1.7 | 14 | 1.0 | 5.9 | 409 | 35.1 | 31.7 | 482 | 1.2 |
| ***Endmember:*** | **-** | **-** | **-** | **0** | **16** | **1.1** | **6.1** | **409** | **35.9** | **32.8** | **480** | **~0** |
|  |  |  |  |  |  |  |  |  |  |  |  |  |
| **Bruse Diffuse Venting/Incubators (71°17.916'N, 5°42.228'W)** | | | | | |  |  |  |  |  |  |  |
| GS17-213-ROV25-IGT2 | 565 | 54 | 5.7 | 50.6 | BD | 0.013 | 0.14 | 470 | 12.0 | 12.4 | - | 27.8 |
| GS17-213-ROV25-IGT4 | 565 | 34 | 5.9 | 51.9 | BD | - | 0.07 | 474 | 11.4 | 11.5 | 525 | 28.1 |
|  |  |  |  |  |  |  |  |  |  |  |  |  |
| **Bottom Seawater** | - | -0.4 | 7.8 | 52.6 | 0 | 0 | 0 | 475 | 10.3 | 10.2 | 545 | 28.2 |
| *T_max_ = maximum measured realtime temperature during sampling. mm = mmol/kg fluid; μM = mmol/L; mM = mmol/L* | | | | | | | | | | | | |
