# Supplementary material for: Tailoring Hydrothermal Vent Biodiversity Toward Improved Biodiscovery Using a Novel in situ Enrichment Strategy
Source: Front Microbiol. 2020 Feb 21;11:249. doi: 10.3389/fmicb.2020.00249 (PMC7046548; doi:10.3389/fmicb.2020.00249)
Supplement: TABLE S2 — Sequencing statistics and 16S rRNA read filtering. [file Table_2.DOCX]

**Table S2.** Sequencing statistics and 16S rRNA read filtering.

| **Sample** | **Total reads** | **# short reads (<250bp)** | **Low quality reads** | **chimeras removed** | **Filtered reads** |
| --- | --- | --- | --- | --- | --- |
| CGB7_1 | 59662 | 16446 | 5797 | 4187 | 33232 |
| CGB7_2 | 31138 | 6028 | 3950 | 1024 | 20136 |
| CGB7_3 | 59526 | 16780 | 7695 | 5084 | 29967 |
| CGB8_1 | 75117 | 17406 | 4901 | 8502 | 44308 |
| CGB8_2 | 70688 | 19628 | 7074 | 4512 | 39474 |
| CGB8_3 | 54417 | 14923 | 4320 | 2354 | 32820 |
| CGB6_1 | 64884 | 14976 | 4377 | 12228 | 33303 |
| CGB6_2 | 77408 | 18110 | 4896 | 27553 | 26849 |
| CGB6_3 | 54509 | 11739 | 3991 | 3316 | 35463 |
