# Supplementary material for: Tailoring Hydrothermal Vent Biodiversity Toward Improved Biodiscovery Using a Novel in situ Enrichment Strategy
Source: Front Microbiol. 2020 Feb 21;11:249. doi: 10.3389/fmicb.2020.00249 (PMC7046548; doi:10.3389/fmicb.2020.00249)
Supplement: TABLE S3 — Most abundant OTUs in the unamended sediment samples. Data are shown for each chamber with increasing sediment depth and temperature (CGB8_1, CGB8_2, and CGB8_3) as relative abundance, with corresponding SILVA taxonomic assignment, closest blast hit and environment in the NCBI non-redundant database. [file Table_3.DOCX]

**Table S3.** Most abundant OTUs (>2% relative abundance within one of the chambers in each of the collected *in-situ* incubators) in the unamended sediment samples. Data are shown for each chamber with increasing sediment depth and temperature (CGB8_1, CGB8_2 and CGB8_3) as relative abundance, with corresponding SILVA taxonomic assignment, closest blast hit and environment in the NCBI non-redundant database.

| **OTUs** | **CGB8_1** | **CGB8_2** | **CGB8_3** | **Taxonomic affiliation** | **Closest hit (%id)** | **Environment** | **Ref** |  |
| --- | --- | --- | --- | --- | --- | --- | --- | --- |
| OTU_1 | 25.04 | 15.2 | 18.8 | Proteobacteria | Uncultured delta proteobacterium, GUAY_50enr_Bac6 (97) | Hydrothermally influenced sediment, Guaymas Basin | (Holler et al., 2011) |  |
| OTU_10 | 7.7 | 1.3 | 0.07 | Unclassified | Uncultured archaeon (87) | Iheya North hydrothermal field | (Yanagawa et al., 2013) |  |
| OTU_13 | 0.8 | 6.5 | 0.11 | Unclassified | Uncultured prokaryote, OTU1897 (85) | Aarhus Bay sediments | Unpubl |  |
| OTU_2 | 1.2 | 14.9 | 24.5 | Aigarchaeota | Uncultured archaeon, Exp331_INH_31A_80 (100) | Iheya North hydrothermal field | (Yanagawa et al., 2013) |  |
| OTU_21 | 0 | 0.002 | 6.02 | Thermodesulfobacteria | Uncultured archaeon proteobacterium, SH05-CAT-29 (99) | Suiyo Seamount, Izu-Bonin Arc | (Higashi et al., 2004) |  |
| OTU_22 | 0.6 | 1.7 | 2.3 | Planctomycetes | Uncultured bacterium, OTU_17635 (94) | Soil of a tobacco plantation, Xiangxi, China | Unpubl |  |
| OTU_27 | 6.9 | 0 | 0.05 | Firmicutes | Uncultured bacterium, Guaymas BIG B21A21 (100) | Hydrothermal organic-rich sediments | (Callac et al., 2013) |  |
| OTU_29 | 2.02 | 4.7 | 0.09 | Unclassified | Bacterium enrichment culture, HB_92 (90) | Crude oil-contaminated soil | Unpubl |  |
| OTU_4 | 4.9 | 11.7 | 13.8 | Euryarchaeota | Uncultured archaeon, GB-EvMd-ANME-1a-C (96) | Hydrothermal Sediment of the Guaymas Basin | (Lever and Teske, 2015) |  |
| OTU_41 | 0.7 | 4.2 | 0.012 | Bathyarchaeota | Uncultured archaeon, WOR1-52_54-21 (99) | Anoxic estuarine sediments | Unpubl |  |
| OTU_6 | 1.5 | 5.9 | 7.06 | Acetothermia | Uncultured bacterium clone PM11 (99) | Shallow hydrothermal, Mexican Pacific West coast | Unpubl |  |
| OTU_7 | 2.7 | 0 | 0 | Bacteroidetes | Uncultured bacterium, BMS3BB04 (99) | Sulfide deposits, Southern Mariana Trough | (Kato et al., 2015) |  |
| OTU_9 | 0.09 | 4.1 | 4.6 | Aigarchaeota | Uncultured archaeon, Fhm3A18 (99) | Deep-sea hydrothermal fields, Southern  Mariana Trough | (Kato et al., 2010) |  |

**References:**

Callac, N., Rommevaux-Jestin, C., Rouxel, O., Lesongeur, F., Liorzou, C., Bollinger, C., Ferrant, A. & Godfroy, A. (2013). Microbial colonization of basaltic glasses in hydrothermal organic-rich sediments at Guaymas Basin. *Frontiers in Microbiology,* 4. DOI 10.3389/fmicb.2013.00250

Higashi, Y., Sunamura, M., Kitamura, K., Nakamura, K.-I., Kurusu, Y., Ishibashi, J.-I., Urabe, T. & Maruyama, A. (2004). Microbial diversity in hydrothermal surface to subsurface environments of Suiyo Seamount, Izu-Bonin Arc, using a catheter-type in situ growth chamber. *FEMS Microbiology Ecology,* 47**:** 327-336. DOI 10.1016/S0168-6496(04)00004-2

Holler, T., Widdel, F., Knittel, K., Amann, R., Kellermann, M.Y., Hinrichs, K.-U., Teske, A., Boetius, A. & Wegener, G. (2011). Thermophilic anaerobic oxidation of methane by marine microbial consortia. *The Isme Journal,* 5**:** 1946. DOI 10.1038/ismej.2011.77

Kato, S., Ikehata, K., Shibuya, T., Urabe, T., Ohkuma, M. & Yamagishi, A. (2015). Potential for biogeochemical cycling of sulfur, iron and carbon within massive sulfide deposits below the seafloor. *Environmental Microbiology,* 17**:** 1817-1835. DOI 10.1111/1462-2920.12648

Kato, S., Takano, Y., Kakegawa, T., Oba, H., Inoue, K., Kobayashi, C., Utsumi, M., Marumo, K., Kobayashi, K., Ito, Y., Ishibashi, J.-I. & Yamagishi, A. (2010). Biogeography and Biodiversity in Sulfide Structures of Active and Inactive Vents at Deep-Sea Hydrothermal Fields of the Southern Mariana Trough. *Applied and Environmental Microbiology,* 76**:** 2968-2979. DOI 10.1128/aem.00478-10

Lever, M.A. & Teske, A.P. (2015). Diversity of Methane-Cycling Archaea in Hydrothermal Sediment Investigated by General and Group-Specific PCR Primers. *Applied and Environmental Microbiology,* 81**:** 1426-1441. DOI 10.1128/aem.03588-14

Yanagawa, K., Nunoura, T., Mcallister, S., Hirai, M., Breuker, A., Brandt, L., House, C., Moyer, C., Birrien, J.-L., Aoike, K., Sunamura, M., Urabe, T., Mottl, M. & Takai, K. (2013). The first microbiological contamination assessment by deep-sea drilling and coring by the D/V Chikyu at the Iheya North hydrothermal field in the Mid-Okinawa Trough (IODP Expedition 331). *Frontiers in Microbiology,* 4. DOI 10.3389/fmicb.2013.00327
