# Supplementary material for: Tailoring Hydrothermal Vent Biodiversity Toward Improved Biodiscovery Using a Novel in situ Enrichment Strategy
Source: Front Microbiol. 2020 Feb 21;11:249. doi: 10.3389/fmicb.2020.00249 (PMC7046548; doi:10.3389/fmicb.2020.00249)
Supplement: TABLE S4 — Most abundant OTUs in BALITM pre-treated spruce sample. Data are shown for each chamber with increasing sediment depth and temperature (CGB6_1, CGB6_2, and CGB6_3) as relative abundance, with corresponding SILVA taxonomic assignment, closest blast hit and environment in the NCBI non-redundant database. [file Table_4.DOCX]

**Table S4.** Most abundant OTUs (>2% relative abundance within one of the chambers in each of the collected *in-situ* incubators) in BALI^TM^ pre-treated spruce sample. Data are shown for each chamber with increasing sediment depth and temperature (CGB6_1, CGB6_2 and CGB6_3) as relative abundance, with corresponding SILVA taxonomic assignment, closest blast hit and environment in the NCBI non-redundant database.

| **OTUs** | **CGB6_1** | **CGB6_2** | **CGB6_3** | **Taxonomic affiliation** | **Closest hit (%id)** | **Environment** | **Ref** |
| --- | --- | --- | --- | --- | --- | --- | --- |
| OTU_7 | 20.5 | 0.01 | 0 | Bacteroidetes | Uncultured bacterium, BMS3BB04 (99) | Sulfide deposits, Southern Mariana Trough | (Kato et al., 2015) |
| OTU_14 | 14.1 | 0 | 0 | Unclassified | Uncultured bacterium, AMSMV-20-B10 (91) | Deep-sea mud volcano, East Mediterranean Sea | (Pachiadaki et al., 2011) |
| OTU_18 | 10.3 | 0 | 0.002 | Deferribacteres | Uncultured bacterium, BAC2_17 (99) | Hydrothermal Sediments, Guaymas Basin | (Dowell et al., 2016) |
| OTU_17 | 9.1 | 2.1 | 0 | Thermotogae | Uncultured bacterium, p816_b_1.02 (94) | Hydrothermal sediments, Southern Okinawa trough | (Nunoura et al., 2010) |
| OTU_12 | 8.2 | 4.4 | 0 | Planctomycetes | Uncultured bacterium Plate5_4664_0_2_16S_BAC_C9Full (96) | Deep-sea coral community | Unpubl |
| OTU_23 | 6.6 | 0 | 0 | Deferribacteres | Uncultured bacterium, HL2S9 (100) | Rhizosphere of *Haloxylon recurvum* | Unpubl |
| OTU_25 | 3.8 | 1.1 | 0 | Spirochaetae | Uncultured bacterium, Smmcs007U (97) | Deep-sea hydrothermal, suiyo seamount | (Kato et al., 2013) |
| OTU_35 | 3.6 | 0 | 0 | Bacteroidetes | Uncultured Bacteroidetes,  pMARB19_24 (99) | Deep sea hydrothermal vent field in Mid-Atlantic Ridge | Unpubl |
| OTU_252 | 2.3 | 0 | 0 | Deferribacteres | Uncultured bacterium, HL2S9 (96) | Rhizosphere of Haloxylon recurvum | Unpubl |
| OTU_32 | 1.4 | 3.6 | 0 | Proteobacteria | Uncultured bacterium, GUAY_37enr_Bac84 (93) | Hydrothermal sediments, Guaymas Basin | (Kellermann et al., 2012) |
| OTU_2 | 0.4 | 0.03 | 11.2 | Aigarchaeota | Uncultured archaeon, Exp331_INH_31A_80 (100) | Iheya North hydrothermal field | (Yanagawa et al., 2013) |
| OTU_1 | 0.4 | 1.3 | 7.6 | Proteobacteria | Uncultured delta proteobacterium, GUAY_50enr_Bac6 (97) | Hydrothermally influenced sediment, Guaymas Basin | (Holler et al., 2011) |
| OTU_16 | 0.2 | 10.6 | 0.005 | Proteobacteria | Uncultured bacterium, Butane50_mme_888_Bact (99) | Anaerobic butane oxidizing enrichment, 50 degrees Celsius | Unpubl |
| OTU_584 | 0.2 | 4.6 | 0 | Spirochaetae | Uncultured bacterium, Smmcs007U (97) | Deep-sea hydrothermal, suiyo seamount | (Kato et al., 2013) |
| OTU_28 | 0.2 | 7.3 | 0.3 | Deferribacteres | *Caldithrix abyssi* DSM 13497 (99) | Mid Atlantic Ridge, Logachev | (Kublanov et al., 2017) |
| OTU_325 | 0.1 | 6.6 | 0 | Proteobacteria | Uncultured bacterium, Butane50_mme_888_Bact (98) | Anaerobic butane oxidizing enrichment, 50 degrees Celsius | Unpubl |
| OTU_11 | 0.04 | 12 | 4.8 | Thermotogae | Thermotogae bacterium enrichment culture, base_BAC_32 (94) | High temperature oil reservoir | Unpubl |
| OTU_499 | 0.04 | 3 | 0 | Proteobacteria | Uncultured bacterium, Butane50_mme_888_Bact (99) | Anaerobic butane oxidizing enrichment, 50 degrees Celsius | Unpubl |
| OTU_5 | 0.03 | 6.1 | 19.5 | Unclassified | Uncultured bacterium, OTU4639 (88) | Sediments of a eutrophic shallow lake | Unpubl |
| OTU_31 | 0.02 | 5.7 | 0 | Acidobacteria | Uncultured bacterium,p816_b_3.16 (98) | Hydrothermal sediments, Southern Okinawa trough | (Nunoura et al., 2010) |
| OTU_26 | 0 | 4.9 | 0 | Unclassified | Uncultured archaeon DGGE gel band 6 (91) | Sulfidic monimolimnion of Ace Lake (Antarctica) | (Coolen et al., 2004) |
| OTU_3 | 0 | 0.4 | 23.6 | Thermotogae | Uncultured bacterium, PNG_War_B170 (99) | Alkaline hot springs of Ambitle Island | (Meyer-Dombard and Amend, 2014) |
| OTU_19 | 0 | 0.01 | 4.4 | Proteobacteria | Uncultured bacterium clone D15_12_SS_A_29 (98) | Coastal waters of the South china sea | (Du et al., 2013) |
| OTU_53 | 0 | 0 | 5.1 | Aigarchaeota | Uncultured archaeon  Smmcs012A (99) | Deep-sea hydrothermal field of the Suiyo Seamount | (Kato et al., 2012) |
| OTU_36 | 0 | 0.07 | 2.2 | Bathyarcheota | Uncultured archaeon, 4559-4-C2-95 (99) | Guaymas Basin hydrothermal vent field | Unpubl |

**References:**

Coolen, M.J.L., Hopmans, E.C., Rijpstra, W.I.C., Muyzer, G., Schouten, S., Volkman, J.K. & Sinninghe Damsté, J.S. (2004). Evolution of the methane cycle in Ace Lake (Antarctica) during the Holocene: response of methanogens and methanotrophs to environmental change. *Organic Geochemistry,* 35**:** 1151-1167. DOI <https://doi.org/10.1016/j.orggeochem.2004.06.009>

Dowell, F., Cardman, Z., Dasarathy, S., Kellermann, M.Y., Lipp, J.S., Ruff, S.E., Biddle, J.F., Mckay, L.J., Macgregor, B.J., Lloyd, K.G., Albert, D.B., Mendlovitz, H., Hinrichs, K.-U. & Teske, A. (2016). Microbial Communities in Methane- and Short Chain Alkane-Rich Hydrothermal Sediments of Guaymas Basin. *Frontiers in Microbiology,* 7. DOI 10.3389/fmicb.2016.00017

Du, J., Xiao, K., Li, L., Ding, X., Liu, H., Lu, Y. & Zhou, S. (2013). Temporal and Spatial Diversity of Bacterial Communities in Coastal Waters of the South China Sea. *PLOS ONE,* 8**:** e66968. DOI 10.1371/journal.pone.0066968

Holler, T., Widdel, F., Knittel, K., Amann, R., Kellermann, M.Y., Hinrichs, K.-U., Teske, A., Boetius, A. & Wegener, G. (2011). Thermophilic anaerobic oxidation of methane by marine microbial consortia. *The Isme Journal,* 5**:** 1946. DOI 10.1038/ismej.2011.77

Kato, S., Ikehata, K., Shibuya, T., Urabe, T., Ohkuma, M. & Yamagishi, A. (2015). Potential for biogeochemical cycling of sulfur, iron and carbon within massive sulfide deposits below the seafloor. *Environmental Microbiology,* 17**:** 1817-1835. DOI 10.1111/1462-2920.12648

Kato, S., Nakawake, M., Kita, J., Yamanaka, T., Utsumi, M., Okamura, K., Ishibashi, J.-I., Ohkuma, M. & Yamagishi, A. (2013). Characteristics of Microbial Communities in Crustal Fluids in a Deep-Sea Hydrothermal Field of the Suiyo Seamount. *Frontiers in Microbiology,* 4. DOI 10.3389/fmicb.2013.00085

Kato, S., Nakawake, M., Ohkuma, M. & Yamagishi, A. (2012). Distribution and phylogenetic diversity of cbbM genes encoding RubisCO form II in a deep-sea hydrothermal field revealed by newly designed PCR primers. *Extremophiles,* 16**:** 277-283. DOI 10.1007/s00792-011-0428-6

Kellermann, M.Y., Wegener, G., Elvert, M., Yoshinaga, M.Y., Lin, Y.-S., Holler, T., Mollar, X.P., Knittel, K. & Hinrichs, K.-U. (2012). Autotrophy as a predominant mode of carbon fixation in anaerobic methane-oxidizing microbial communities. *Proceedings of the National Academy of Sciences,* 109**:** 19321-19326. DOI 10.1073/pnas.1208795109

Kublanov, I.V., Sigalova, O.M., Gavrilov, S.N., Lebedinsky, A.V., Rinke, C., Kovaleva, O., Chernyh, N.A., Ivanova, N., Daum, C., Reddy, T.B.K., Klenk, H.-P., Spring, S., Göker, M., Reva, O.N., Miroshnichenko, M.L., Kyrpides, N.C., Woyke, T., Gelfand, M.S. & Bonch-Osmolovskaya, E.A. (2017). Genomic Analysis of Caldithrix abyssi, the Thermophilic Anaerobic Bacterium of the Novel Bacterial Phylum Calditrichaeota. *Frontiers in Microbiology,* 8. DOI 10.3389/fmicb.2017.00195

Meyer-Dombard, D.a.R. & Amend, J.P. (2014). Geochemistry and microbial ecology in alkaline hot springs of Ambitle Island, Papua New Guinea. *Extremophiles,* 18**:** 763-778. DOI 10.1007/s00792-014-0657-6

Nunoura, T., Oida, H., Nakaseama, M., Kosaka, A., Ohkubo, S.B., Kikuchi, T., Kazama, H., Hosoi-Tanabe, S., Nakamura, K.-I., Kinoshita, M., Hirayama, H., Inagaki, F., Tsunogai, U., Ishibashi, J.-I. & Takai, K. (2010). Archaeal Diversity and Distribution along Thermal and Geochemical Gradients in Hydrothermal Sediments at the Yonaguni Knoll IV Hydrothermal Field in the Southern Okinawa Trough. *Applied and Environmental Microbiology,* 76**:** 1198-1211. DOI 10.1128/aem.00924-09

Pachiadaki, M.G., Kallionaki, A., Dählmann, A., De Lange, G.J. & Kormas, K.A. (2011). Diversity and Spatial Distribution of Prokaryotic Communities Along A Sediment Vertical Profile of A Deep-Sea Mud Volcano. *Microbial Ecology,* 62**:** 655-668. DOI 10.1007/s00248-011-9855-2

Yanagawa, K., Nunoura, T., Mcallister, S., Hirai, M., Breuker, A., Brandt, L., House, C., Moyer, C., Birrien, J.-L., Aoike, K., Sunamura, M., Urabe, T., Mottl, M. & Takai, K. (2013). The first microbiological contamination assessment by deep-sea drilling and coring by the D/V Chikyu at the Iheya North hydrothermal field in the Mid-Okinawa Trough (IODP Expedition 331). *Frontiers in Microbiology,* 4. DOI 10.3389/fmicb.2013.00327
