# Supplementary material for: Tailoring Hydrothermal Vent Biodiversity Toward Improved Biodiscovery Using a Novel in situ Enrichment Strategy
Source: Front Microbiol. 2020 Feb 21;11:249. doi: 10.3389/fmicb.2020.00249 (PMC7046548; doi:10.3389/fmicb.2020.00249)
Supplement: TABLE S5 — Most abundant OTUs in the Salmeal sample. Data are shown for each chamber with increasing sediment depth and temperature (CGB7_1, CGB7_2, and CGB7_3) as relative abundance, with corresponding SILVA taxonomic assignment, closest blast hit and environment in the NCBI non-redundant database. [file Table_5.DOCX]

**Table S5**. Most abundant OTUs (>2% relative abundance within one of the chambers in each of the collected *in-situ* incubators) in the salmeal sample. Data are shown for each chamber with increasing sediment depth and temperature (CGB7_1, CGB7_2 and CGB7_3) as relative abundance, with corresponding SILVA taxonomic assignment, closest blast hit and environment in the NCBI non-redundant database.

| **OTUs** | **CGB7_1** | **CGB7_2** | **CGB7_3** | **Taxonomic affiliation** | **Closest hit NCBI nr (%id)** | **Environment** | | **Ref** |
| --- | --- | --- | --- | --- | --- | --- | --- | --- |
| OTU_6 | 20.5 | 10.5 | 4.8 | Acetothermia | Uncultured bacterium clone PM11 (99) | | Shallow hydrothermal, Mexican Pacific West coast | Unpubl |
| OTU_2 | 11.1 | 0.6 | 1.4 | Aigarchaeota | Uncultured archaeon, Exp331_INH_31A_80 (100) | | Iheya North hydrothermal field | (Yanagawa et al., 2013) |
| OTU_3 | 6 | 0.01 | 0.07 | Thermotogae | Uncultured bacterium, PNG_War_B170 (99) | | Alkaline hot springs of Ambitle Island | (Meyer-Dombard and Amend, 2014) |
| OTU_9 | 5.6 | 0.1 | 1.2 | Aigarchaeota | Uncultured archaeon, Fhm3A18 (99) | | Deep-sea hydrothermal fields, Southern  Mariana Trough | (Kato et al., 2010) |
| OTU_8* | 4.9 | 21.4 | 13.4 | Unclassified | Uncultured euryarchaeote clone M08_80E2A  (88) | | Hydrothermal chimney Lucky Strike vent MAR | Unpubl |
| OTU_40 | 3.4 | 0 | 0.05 | Unclassified | Uncultured microorganism, SZY.L801.7_Tag14738 (94) | | Oil field | Unpubl |
| OTU_24 | 3 | 2.8 | 2.1 | Thermotogae | Thermotoga sp. FC203 (94) | | Fang hot spring | Unpubl |
| OTU_54 | 2.7 | 0 | 0.1 | Aminicenantes | Uncultured bacterium a2b010 (99) | | Hydrothermal sediments, Guaymas Basin | (Teske et al., 2002) |
| OTU_93 | 2.3 | 29.5 | 1.9 | Aigarchaeota | Uncultured archaeon,  SSM040-10 (98) | | Deep-sea hydrothermal fluid in the Suiyo Seamount | (Kimura et al., 2010) |
| OTU_46 | 2.1 | 1.8 | 1.7 | Korarchaeota | Uncultured archaeon, HL-H_14-17_arc80 (99) | | Shallow hydrothermal vent, Hot Lake | Unpubl |
| OTU_47 | 2.1 | 0 | 0 | Proteobacteria | Uncultured bacterium, Guaymas BIG B11B1 (94) | | Hydrothermal  organic-rich sediments, Guaymas Basin | (Callac et al., 2013) |
| OTU_42 | 2 | 0.1 | 0.2 | Euryarchaeota | Uncultured microorganism, GJ0EXAY04I7C7I (99) | | Middle Valley hydrothermal vent sediment | (Wankel et al., 2012) |
| OTU_1 | 1.7 | 0.3 | 5.2 | Proteobacteria | Uncultured delta proteobacterium, GUAY_50enr_Bac6 (97) | | Hydrothermally influenced sediment, Guaymas Basin | (Holler et al., 2011) |
| OTU_20 | 1.5 | 0.2 | 2.8 | Proteobacteria | Uncultured bacterium clone T3-1_199 (100) | | Deep-sea hydrothermal sediment, Okinawa Trough | (Wang et al., 2018) |
| OTU_15 | 0.5 | 1.8 | 14.8 | Aquificae | Thermosulfidibacter takaii ABI70S6 (97) | | Deep-sea hydrothermal field, Southern Okinawa Trough | (Nunoura et al., 2008) |
| OTU_6 | 0.3 | 1.2 | 2.2 | Aigarchaeota | Uncultured archaeon, 1A-52 (100) | | Active Sulfide Chimney, Endeavour Segment of Juan de Fuca Ridge | Unpubl |
| OTU_37 | 0.2 | 3.6 | 2.4 | Euryarchaeota | Uncultured bacterium,  Fhm2A94 (100) | | Deep-sea hydrothermal fields, Southern Mariana Trough | (Kato et al., 2010) |
| OTU_30* | 0.1 | 7.2 | 9.6 | Crenarchaeota | Uncultured Desulfurococcales archaeon, HTM866S-A1  (99) | | Chimney structure at hydrothermal active area of Hatoma Knoll, Okinawa Trough | (Yoshida-Takashima et al., 2012) |

*Unique OTUs for the CGB7 salmeal incubator

**References:**

Callac, N., Rommevaux-Jestin, C., Rouxel, O., Lesongeur, F., Liorzou, C., Bollinger, C., Ferrant, A. & Godfroy, A. (2013). Microbial colonization of basaltic glasses in hydrothermal organic-rich sediments at Guaymas Basin. *Frontiers in Microbiology,* 4. DOI 10.3389/fmicb.2013.00250

Holler, T., Widdel, F., Knittel, K., Amann, R., Kellermann, M.Y., Hinrichs, K.-U., Teske, A., Boetius, A. & Wegener, G. (2011). Thermophilic anaerobic oxidation of methane by marine microbial consortia. *The Isme Journal,* 5**:** 1946. DOI 10.1038/ismej.2011.77

Kato, S., Takano, Y., Kakegawa, T., Oba, H., Inoue, K., Kobayashi, C., Utsumi, M., Marumo, K., Kobayashi, K., Ito, Y., Ishibashi, J.-I. & Yamagishi, A. (2010). Biogeography and Biodiversity in Sulfide Structures of Active and Inactive Vents at Deep-Sea Hydrothermal Fields of the Southern Mariana Trough. *Applied and Environmental Microbiology,* 76**:** 2968-2979. DOI 10.1128/aem.00478-10

Kimura, H., Mori, K., Tashiro, T., Kato, K., Yamanaka, T., Ishibashi, J.-I. & Hanada, S. (2010). Culture-Independent Estimation of Optimal and Maximum Growth Temperatures of Archaea in Subsurface Habitats Based on the G+C Content in 16S rRNA Gene Sequences. *Geomicrobiology Journal,* 27**:** 114-122. DOI 10.1080/01490450903456699

Meyer-Dombard, D.a.R. & Amend, J.P. (2014). Geochemistry and microbial ecology in alkaline hot springs of Ambitle Island, Papua New Guinea. *Extremophiles,* 18**:** 763-778. DOI 10.1007/s00792-014-0657-6

Nunoura, T., Oida, H., Miyazaki, M. & Suzuki, Y. (2008). Thermosulfidibacter takaii gen. nov., sp. nov., a thermophilic, hydrogen-oxidizing, sulfur-reducing chemolithoautotroph isolated from a deep-sea hydrothermal field in the Southern Okinawa Trough. *International Journal of Systematic and Evolutionary Microbiology,* 58**:** 659-665. DOI doi:10.1099/ijs.0.65349-0

Teske, A., Hinrichs, K.U., Edgcomb, V., De Vera Gomez, A., Kysela, D., Sylva, S.P., Sogin, M.L. & Jannasch, H.W. (2002). Microbial diversity of hydrothermal sediments in the Guaymas Basin: evidence for anaerobic methanotrophic communities. *Appl Environ Microbiol,* 68**:** 1994-2007.

Wang, L., Yu, M., Liu, Y., Liu, J., Wu, Y., Li, L., Liu, J., Wang, M. & Zhang, X.-H. (2018). Comparative analyses of the bacterial community of hydrothermal deposits and seafloor sediments across Okinawa Trough. *Journal of Marine Systems,* 180**:** 162-172. DOI <https://doi.org/10.1016/j.jmarsys.2016.11.012>

Wankel, S.D., Adams, M.M., Johnston, D.T., Hansel, C.M., Joye, S.B. & Girguis, P.R. (2012). Anaerobic methane oxidation in metalliferous hydrothermal sediments: influence on carbon flux and decoupling from sulfate reduction. *Environmental Microbiology,* 14**:** 2726-2740. DOI 10.1111/j.1462-2920.2012.02825.x

Yanagawa, K., Nunoura, T., Mcallister, S., Hirai, M., Breuker, A., Brandt, L., House, C., Moyer, C., Birrien, J.-L., Aoike, K., Sunamura, M., Urabe, T., Mottl, M. & Takai, K. (2013). The first microbiological contamination assessment by deep-sea drilling and coring by the D/V Chikyu at the Iheya North hydrothermal field in the Mid-Okinawa Trough (IODP Expedition 331). *Frontiers in Microbiology,* 4. DOI 10.3389/fmicb.2013.00327

Yoshida-Takashima, Y., Nunoura, T., Kazama, H., Noguchi, T., Inoue, K., Akashi, H., Yamanaka, T., Toki, T., Yamamoto, M., Furushima, Y., Ueno, Y., Yamamoto, H. & Takai, K. (2012). Spatial Distribution of Viruses Associated with Planktonic and Attached Microbial Communities in Hydrothermal Environments. *Applied and Environmental Microbiology,* 78**:** 1311-1320. DOI 10.1128/aem.06491-11
